# Supplementary material for: Fermentation Conditions Affect the Synthesis of Volatile Compounds, Dextran, and Organic Acids by Weissella confusa A16 in Faba Bean Protein Concentrate
Source: Foods. 2022 Nov 10;11(22):3579. doi: 10.3390/foods11223579 (PMC9689515; doi:10.3390/foods11223579)
Supplement: Supplementary file 1 [file foods-11-03579-s001.zip › foods-1973681-supplementary.pdf]

Table S1. Measured and reference Linear Retention Indexes.

Volatile compounds	Measured LRI	Reference LRI	
		Column SPB-624[a]	Column OV17[b]
**Organic Acids**			
Acetic acid	752	-	809
Isovaleric acid	959	-	1040
**Alcohols**			
Ethanol	3.56**	-	-
3-Methyl-1-butanol	813	-	844
1-Pentanol	845	822	850
1-Penten-3-ol	756	-	-
3-Hexen-1-ol	939	-	-
1-Hexanol	947	920	990
1-Octanol	816	1120	1072
**Aldehydes**			
3-Methylbutanal	717	-	-
Pentanal	759	734	767
Hexanal	865	840	881
2-Hexenal	936	907	957
Heptanal	971	939	985
2-Heptenal	1045	1005	-
2-Octenal	1152	1115	1151
Nonanal	1182	1152	1195
**Alkanes**			
Hexane	620	600	-
Octane	823	800	-
**Esters**			
Ethyl acetate	664	-	719
Isoamyl acetate	931	-	938
Acetic acid methyl ester	4.49**	-	-
Acetic acid hexyl ester	1070	-	1084
Hexanoic acid ethyl ester	1054	-	1054
Heptanoic acid ethyl ester	1156	-	-
Octanoic acid ethyl ester	1257	-	1260
Isoamyl valerate	1163	-	-
Methyl isovalerate	827	-	-
Ethyl isovalerate	903	-	-
Propyl isovalerate	1202	-	-
Ethyl lactate	889	-	1101
**Ketones**			
Acetoin	805	-	809
2-Hexanone	859	-	-
3-Hexanone	851	-	-
**Aromatic Compounds**			
2-Methylfuran	655	-	-
2-Pentylfuran	1039	1003	-
**Terpenes**			
Alpha-pinene	975	-	945
Delta-3-carene	1056	-	1100
D-Limonene	1077	-	1056

LRI, Linear Retention Index; **, retention time (LRI not determined because the compounds eluted before the retention time of hexane (retention time 4.8 min); [a], source: Paradiso et al. [60]; [b], source: flavornet.org [61]; –, not determined.
